# Are Genetic and Environmental Contributions to Verbal Fluency and Episodic Memory Solely Moderated by Sleep?

**DOI:** 10.1093/geroni/igab046.3563

**Published:** 2021-12-17

**Authors:** Tina Vo, Shandell Pahlen, William Kremen, Matthew Mc Gue, Marianne Nygaard, Chandra Reynolds

**Affiliations:** 1 University of California Riverside, Riverside, California, United States; 2 University of California, University of California, California, United States; 3 University of California San Diego, La Jolla, California, United States; 4 University of Minnesota, Minneapolis, Minnesota, United States; 5 Department of Public Health, University of Southern Denmark, Odense, Syddanmark, Denmark

## Abstract

Decreases in sleep duration and cognitive functioning often occur and co-occur in aging although these patterns are not universal. Underlying etiologies, i.e., genetic and environmental factors, contribute to why people differ on cognitive functioning at shorter versus longer sleep durations. The current study tested whether sleep duration alters the genetic and environmental contributions to why middle-aged and older adults vary on cognitive functioning. Using 4 twin studies from the Interplay of Genes and Environment Across Multiple Studies (IGEMS) consortium (Mage=56.5, range=35.0-91.2, N=5,210, 1,083 complete MZ pairs, 1,522 complete DZ pairs) we tested quantitative genetic twin models considering sleep, depressive symptoms, and age as moderators of verbal fluency (i.e., Animal Naming) and episodic memory (i.e., Word List). For verbal fluency, sleep duration and depressive symptoms were significant when dropped together from the model (χ2(6)=15.22, p=0.02) but not individually (χ2sleep(3)=7.17, p=0.07; χ2dep(3)=5.81, p=0.12), indicating that both moderators may affect differences in verbal fluency performance. For episodic memory, sleep duration moderation was only significant via the shared environmental factor (χ2(1)=5.26, p=0.02), indicating that sleep may affect differences in episodic memory performance via environmental influences that make siblings more similar to one another. Overall, results illustrate patterns of higher genetic influences on cognitive function at short sleep (4 hours) and higher shared environmental influences on cognitive function at long sleep (10 hours). These findings may align with associations of upregulation of neuroinflammatory processes at short sleep and common reporting of mental fatigue at long sleep, both of which are associated with poorer cognitive functioning.

